# Preparation of Activated Carbon Derived from Jordanian Olive Cake and Functionalized with Cu/Cu_2_O/CuO for Adsorption of Phenolic Compounds from Olive Mill Wastewater

**DOI:** 10.3390/ma14216636

**Published:** 2021-11-04

**Authors:** Muna Abu-Dalo, Jehad Abdelnabi, Abeer Al Bawab

**Affiliations:** 1 Chemistry Department, Jordan University of Science and Technology, Irbid 22110, Jordan; abdelnabi19@yahoo.com; 2 Chemistry Department, School of Science, University of Jordan, Amman 11942, Jordan; 3 Hamdi Mango Center for Scientific Research, University of Jordan, Amman 11942, Jordan

**Keywords:** functionalized activated carbon, olive mill wastewater, olive cake, Cu/Cu_2_O/CuO, adsorption, total phenolic content

## Abstract

Olive oil production generates solid and liquid wastes that cause various environmental problems due to their high phenols and polyphenols load. Although many treatment methods were investigated to manage these wastes, more research is still needed to identify simple and cost-effective approaches. In this study, activated carbon (AC) was prepared from olive cake waste and functionalized with Cu/Cu_2_O/CuO for efficient and selective removal of phenolic content from olive mill wastewater (OMW). AC media were characterized by scanning electron/dispersive X-ray spectroscopy (SEM-EDS), X-ray diffraction (XRD), Fourier transform infrared (FTIR) spectrometry, and Brunauer–Emmett–Teller (BET) surface area analysis. The optimum adsorption parameters were investigated, and the adsorption isotherms, thermodynamics, and kinetics were determined. The adsorption of phenols onto copper oxide AC was best described by the Langmuir adsorption with maximum adsorption capacity of 13.9, 12.7, and 9.9 mg/g at 311, 302, and 293 K, respectively. The adsorption reaction was found to be spontaneous and endothermic where ∆H° and ∆G° were found to be 30.104 kJ/mol and −1.765, −2.839, and −3.723 (kJ/mol) at 311, 302, and 293 K, respectively. In addition, the kinetics data were perfectly fit by the pseudo-second-order model. The activated product derived from recyclable olive cake and enriched with inorganic functionality can offer a cost-effective treatment solution for OMW; thus, reducing both the liquid and solid waste generated from the olive mill industry.

## 1. Introduction

The harvesting of olive trees and the extraction of olive oil has been a traditional practice in the Mediterranean region for more than 7000 years [1]. The conventional press extraction technique as well as the continuous three-phase decanter process generates three products: olive oil (20%) and two streams of waste: a solid waste (30%) called olive cake and an aqueous waste (50%) called olive mill wastewater (OMW). Unfortunately, the management of these wastes is luxurious and delicate task [2].

OMW is one of the robust industrial effluents, due to its strong organic content, high suspended solid content, and high concentration of recalcitrant compounds such as lignins and tannins that give it a typical dark color. It contains phenolic compounds that can be either simple phenols and flavonoids, or polyphenols that formed through the polymerization of simple phenols. Seasonal production of olive oil demands an appropriate and accommodating technology that can be operated in non-continuous mode. Furthermore, olive mills are predominantly small businesses, scattered around the olive production regions, making individual on-site treatment options exorbitant [2]. Deplorably, these mills discharge OMW without any processing because of the lack of legislation and national policy that regulates the management of OMW, lack of awareness, and the requirement for high-cost and sophisticated techniques for treatment processes. Therefore, the only way to dispose of OMW is by transporting it to landfill sites which also is considered a very high-cost approach [3]. In this regard, numerous methods from biological to physical, thermal, chemical, and physicochemical approaches have been investigated to handle OMW [3]. Among these techniques, physicochemical processes particularly adsorption is deemed to be the most appropriate treatment technique since it is considered a cost-effective method that can be performed without the requirement of harsh conditions, sophisticated techniques, or high energy input. Moreover, no toxic fumes or bad odor arise upon its utilization [2].

Activated carbon (AC) acquires attractive properties i.e., selectivity, large surface area, and porosity that empower its application in adsorption. Nevertheless, the high cost of commercial AC limited its applications [4]. The utilization of industrial wastes and agricultural by-products can offer a cost-effective solution [5,6]. Different raw carbonaceous materials were employed to prepare activated carbon, for instance, the timber of pine, shells of coconut [7], and almond [8]. However, these categories of precursors are imported, which increases their cost, making it obligatory to find another available raw material for activated carbon preparation [9]. In addition, numerous convenient lignocellulosic materials counting stones of peach [10], husks of rice [11], and stones of olives [8] have been tested as precursors for AC preparation.

In contrast, olive cake as lignocellulosic material [2] received less attention for the synthesis of AC [9]. The use of olive cake as a feedstock for AC production not only provides a cost-effective adsorbent but also decreases the amounts of solid wastes produced upon olive milling processes. Limited studies were conducted to convert olive cake to AC through physical and chemical activation procedures [12] and utilize it as an adsorbent for heavy metals [5,9,13], phenols, or polyphenols from synthetic wastewater [5,14,15], and dyes [5,16,17]. However, the olive cake has not been used in treating real OMW samples till now.

Surface functionalization can effectively enhance the adsorption capability of AC by improving its properties and selectivity for certain applications. For instance, the functionalization of AC with natural materials such as beetroot extract increases the scavenging capability of AC for dyes three times compared with the parent AC [18]. Polymers functionalization of AC also provides additional active sites that enhance the selectivity of AC for heavy metals, and dyes [19,20]. Immobilization of organic compounds such as Ethylenediaminetetraacetic acid, tetraethylenepentamine, triethylenetetramine, propylenediamine, and ethylenediamine on the surface of AC imported new functional groups responsible for improving heavy metals and dyes removal [21,22,23,24,25]. Alternatively, surface functionalization with inorganic compounds was demonstrated to be effective for organic pollutants adsorption. For instance, cobalt acetate modified AC was very selective for CH_3_SH [26]. Cu(II)/Fe (III) impregnated AC proved its ability for cephalexin adsorption [27], copper oxide functionalized AC enhanced the adsorption of Propanethiol, toluene, and paracetamol [28,29,30], and CuO-ZnO-La_2_O_3_/activated carbon was an efficient adsorbent for phosphine (PH_3_) [31]. However, to the best of our knowledge, this is the first study that investigates the effect of introducing Cu/Cu_2_O/CuO on the adsorption ability of AC derived from olive cake. 

This study aims to explore the feasibility of delivering highly effective activated carbon adsorbent derived from recyclable olive cake and functionalized with Cu/Cu_2_O/CuO for scavenging phenols and polyphenols from OMW. The new prepared adsorbent product can be adopted and utilized by olive-mills owners to reduce olive mill wastes and eliminate their environmental problems. The adsorbent product, enriched in TPC can be recovered by heat treatment, regenerated, and reused for a new treatment cycle.

## 2. Materials and Methods

### 2.1. Adsorbent Preparation and Characterization

#### 2.1.1. Preparation of Activated Carbon (AC)

The olive cake obtained from the olive oil mill (Abuatheih olive press, Balqa, Jordan) was first soaked in deionized water (1 g olive cake:10 mL deionized water) and stirred for 2 h at 50 °C and 800 rpm to remove any adherent dirt, then filtered and dried at 110 °C until reaching a constant weight [12]. The char was subsequently prepared by calcination of olive cake at 800 °C for 1 h in a muffle furnace (Witag, Berlin, Germany) using a tightly closed crucible that inserted inside a bigger crucible, in which the inner space was filled with sand and packed to the top of the crucible then closed tightly with wires to prevent the oxidation of the sample. Then the char was mixed with KOH (Lab. Chemicals, Nottengham, UK) in 1:2 char: KOH weight ratio for 1 h and 800 rpm, then filtered and dried at 110 °C, follow by retaining samples to muffle furnace to be activated at 800 °C for 8 h. The pH of the produced AC was kept between 6.5–7.0 by washing with deionized water and a few drops of HCl (37%, VWR, Radnor, PA, USA) that increase the rate of pH neutralization after base treatment [5,12,32]. 

#### 2.1.2. Functionalization of AC with Cu/Cu_2_O/CuO

AC was functionalized with Cu/Cu_2_O/CuO according to Arianto et al., 2019, with some modifications. Each 1.00 g of AC was oxidized with 10.0 mL of nitric acid (65%, Super Chem, Chennai, India) through reflux at 120 °C for 3 h, subsequently, filtered, washed with deionized water, and dried at 80 °C [30]. Oxidation of AC was carried out to deliver an active site on the AC surface where copper can connect. Oxidized AC (Ox-AC) was then impregnated with 0.2 M aqueous copper nitrate (Scharlau Chemie, Barcelona, Spain) solution (1 g Ox-AC: 100 mL solution) and stirred for 1 h at 600 rpm. Afterward, the precipitate was filtered, washed until the filtrate was colorless, and dried at 80 °C. The product was then calcinated at 950 °C for 5 min in closed crucibles to convert copper nitrate to s mixture of metallic copper and copper oxides. This sample was labeled as Ox-AC/Cu/Cu_2_O/CuO, then ground to particle size < 45 µm to be used for adsorption of total phenolic contents (TPC). 

#### 2.1.3. Characterization of Activated Carbon Samples

The pH drift method was utilized in point of zero charge determination. The pH of 0.1 M NaNO_3_ (Hopkin & Williams, London, UK) solution was adjusted between 2–12 by adding either HCl or NaOH (pure grade, BBC Chemicals, EU). Then, 0.05 g of either AC or Ox-AC or Ox-AC/Cu/Cu_2_O/CuO was added to 25.0 mL of the solution and left shaking at room temperature for 24 h. Subsequently, the final pH was recorded and plotted against the initial pH to determine the point at which the initial and final pH values were equal for each sample [33]. A scanning electronic microscope/dispersive X-ray spectroscope (SEM-EDS) (QUANTA FEG 450, FEI, Hillsboro, OR, USA) was employed for surface imaging and elemental distribution detection. Fourier Transform Infrared (FTIR) spectra were recorded between 4000 and 500 cm^−1^ on an FT-IR spectrometer (Thermo Nicolet NEXUS 670, GMI, Ramsey, MN, USA) with an ATR module. The surface area of the activated carbon samples was measured by N_2_ adsorption–desorption at 77 K using a surface area analyzer (Quantachrome Corporation, 360Engineering, Golden, CO, USA). The X-ray powder diffraction (XRD) patterns were measured on an Ultima IV (Rigaku, Tokyo, Japan) diffraction with Cu X-ray radiation operator/40 kV/20 mA. 

### 2.2. Olive Mill Wastewater (Adsorbate)

Fresh OMW samples were collected during the cultivation period from a three-phase local olive mill (Bilal Olive Press, Amman, Jordan). Subsequently, they were pretreated through acidification with hydrochloric acid to prevent degradation of phenolic compounds [34], and filtration to decrease the suspended solid content. The samples were kept in a dark area to avoid photodegradation. The major physicochemical characteristics of the OMW were analyzed for the raw and pretreated samples, respectively. OMW was diluted using deionized water before the adsorption process as the effect of the initial TPC value was studied. Hence, the samples were diluted to TPC values of 124, 93, and 62 mg/L.

### 2.3. Total Phenol Determination

Folin Ciocalteau colorimetric method was utilized to determine TPC in OMW samples. Gallic acid (99%, Xilong, Guangdong, China) was used as a standard reference compound thus the results were stated as (mg/L) Gallic acid equivalent. According to Abdelnabi, 2019 procedure; the pH of OMW samples was adjusted to 2.0 prior to TPC measurements. Subsequently, 2.0 mL of each sample was defatted with 1:1 *v*:*v* hexane (95%, ACS, Point Pleasant, NJ, USA) then extracted twice with 1:1 *v*:*v* ethyl acetate (99.8%, Carbon Group, Cork, Ireland) that was collected and dried using a rotary evaporator (RE 300, MESLO, Nicosia, Cyprus). Afterward, the residue was dissolved in 10 mL methanol (HPLC grade, ACS, Point Pleasant, NJ, USA) and used in the subsequent steps. 1 mL of either OMW extracted samples, standard Gallic acid solution (100, 200, 300, 400, and 500 mg/L), or blank solution (deionized water) was added to 9.0 mL of deionized water in a 25.0 mL volumetric flask. Then 1.0 mL of folin (Merck, Darmstadt, Germany) followed by 10.0 mL of 7% by weight Na_2_CO_3_ (AR grade, SDFCL, Tamil Nadu, India) were added and completed to the mark with deionized water. The samples were mixed and kept in dark for 90 min before the absorbance of each sample was measured against the blank using a spectrophotometer (SpectroDirect-Lovibond single-beam, Amesbury, UK) at 750 nm [33]. 

### 2.4. Adsorption Experiments for Ox-AC/C/Cu_2_O/CuO

**Figure 1:**
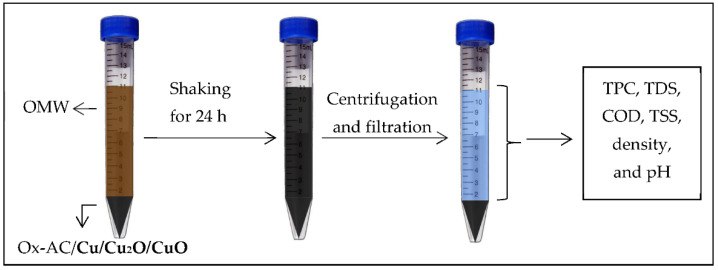
A schematic diagram for the adsorption process.

To test the capacity of Ox-AC/Cu/Cu_2_O/CuO to phenolic compounds, adsorption experiments were conducted by mixing the appropriate dose of the adsorbent with OMW whose pH was adjusted to the desired value and left shacking on the water path shaker (KÖTTERMAN 3047, Uetze, Germany) until the equilibrium is reached. The adsorbent was then separated by centrifugation at 5300 rpm for 30 min followed by filtration using syringe filters (0.25 µm) (Labfil, Zhejiang, China). OMW samples were then subjected to a TPC test as presented in Figure 1. 

#### 2.4.1. Influence of the Adsorbent Concentration 

The influence of the Ox-AC/Cu/Cu_2_O/CuO dose in the adsorption was examined using three different doses specifically 1%, 3%, and 5% by wt., at five different contact times namely 1, 3, 6, 24, and 48 h. The other parameters were held constant; the temperature and the pH were 295 K, and 5.0, respectively. The initial TPC concentration was 124 mg/L.

#### 2.4.2. Influence of the pH

To achieve the optimum pH value, adsorption experiments were conducted at different OMW pH values 2.0, 5.0, 8.0, and 11.0 adjusted using 1 M HCl and 1 M NaOH at a constant temperature of 295 K, initial TPC concentration of 124 mg/L, adsorbent dose of 1% by wt., and contact time of 24 h.

#### 2.4.3. Influence of Initial TPC

To study the influence of the initial TPC concentration, 1% by wt. Ox-AC/Cu/Cu_2_O/CuO was added to OMW with different initial TPC concentrations of 124, 93, and 62 mg/L and kept shaking at a constant temperature, and pH of 295 K, and 5.0, respectively for different contact times of 0.5, 1, 2, 3, 4, 24 and 48 h. 

#### 2.4.4. Influence of Temperature 

The temperature effect on adsorption was tested at various temperatures of 293, 302, and 311 K and different initial TPC of 124, 93, and 62 mg/L, utilizing a 1% dose of Ox-AC/Cu/Cu_2_O/CuO, pH 5.0, and contact time 24 h.

#### 2.4.5. Influence of Ionic Strength

To test the influence of ionic strength on the adsorption process, different amounts of NaCl (extra pure, Lobachemie, India), i.e., 0.05, 0.10, and 0.20 g were added to OMW (initial TPC was 124 mg/L), the Ox-AC/Cu/Cu_2_O/CuO dose was 1% by wt. and the pH and temperature were kept constant at 5.0 and 295 K, respectively.

### 2.5. Modeling of Thermodynamic, Kinetic and Adsorption Isotherms

The amount of TPC uptake at any time $\left(q_{t}\right)$ per unit mass of Ox-AC/Cu/Cu_2_O/CuO in (mg/g) is evaluated using Equation (1):

(1) $$q_{t}=\frac{\left(C_{0}-C_{t}\right)V}{m}$$

where $C_{0}$ is the initial TPC concentration and $C_{t}$ is the TPC at any time, in mg/L, V is the volume of sample in (L) and m is the mass of Ox-AC/Cu/Cu_2_O/CuO in (g). Meanwhile, the TPC uptake at equilibrium $(q_{e}$) is determined by Equation (2):

(2) $$q_{e}=\frac{\left(C_{0}-C_{e}\right)V}{m}$$

where $C_{\text{e}}$ is the TPC concentration at equilibrium.

Modeling of the adsorption kinetics was achieved using the pseudo-first-order model in Equation (3), pseudo-second-order model in Equation (4), and Intra-particle diffusion model, given by Equation (5):

(3) $$\ln\left(q_{e}-q_{t}\right)=\ln q_{e}-k_{1}t$$

(4) $$\frac{t}{q_{t}}=\frac{1}{k_{2}\;q_{e}}+\frac{t}{q_{e}}$$

(5) $$q_{t}=k_{p}\sqrt{t}+C$$

where $k_{1}$ (h^−1^), $k_{2}$ (g/mg·h), and k_p_ (g/mg·h^0.5^) are the equilibrium rate constants of first order, second, and Intra-particle diffusion adsorption reactions, respectively, and C is constant [35,36].

To identify the surface characteristics of the adsorbent; adsorption data are typically modeled by one of the well-known isotherms such as Langmuir, and Freundlich, by correlating the equilibrium concentration of the adsorbate with that on the adsorbent surface. In this study, adsorption data were fitted to Langmuir and Freundlich’s isotherms described by Equations (6) and (7), respectively.

(6) $$\frac{1}{q_{e}}=\frac{1}{Q_{m}}+\left(\frac{1}{C_{e}}\times \frac{1}{b\times Q_{m}}\right)$$

(7) $$\ln q_{e}={\ln K}_{F}+\frac{1}{n}{\ln C}_{e}$$

where $Q_{m}$ and b are factors of the Langmuir adsorption isotherm; associated with the maximum theoretical adsorption capacity and energy, respectively. However, $k_{F}$ is the Freundlich constant related to the adsorption capacity, and $\frac{1}{n}$ is related to the intensity of adsorption [37,38]. 

The adsorption thermodynamic parameters: Enthalpy (ΔH°), Gibbs free energy (ΔG°), and Entropy (ΔS°) were evaluated by the following equations [39]:

(8) ΔG°=−RT ln(Kc)

(9) $$\ln\mathrm{Kc}=\frac{\Delta S{}^{\circ}}{R}-\frac{\Delta H}{\mathrm{RT}}$$

(10) $$\mathrm{Kc}=\frac{C_{0}-C_{e}}{C_{e}}$$

## 3. Results and Discussion

### 3.1. Characterization of Activated Carbon

#### 3.1.1. Scanning Electron Microscopy Coupled with Dispersive X-ray Spectroscopy (SEM-EDS) Characterization

**Figure 2:**
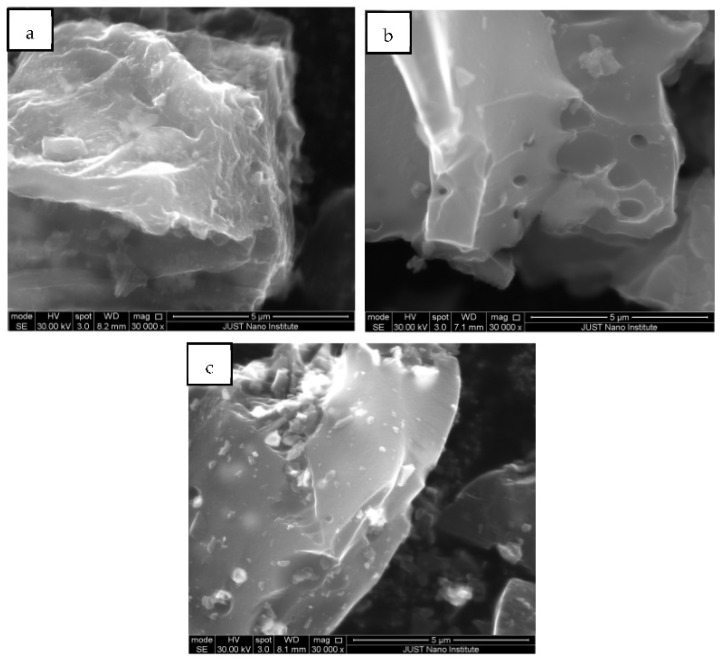
SEM images for (**a**) parent AC, (**b**) Ox-AC, and (**c**) Ox-AC/Cu/Cu_2_O/CuO.

To emphasize the morphological changes that occurred for the media surfaces and the distribution of chemical elements before and after functionalization, SEM-EDS characterization was performed for the parent AC, Ox-AC, and Ox-AC/Cu/Cu_2_O/CuO as shown in Figure 2 and Table 1. Results showed that the parent AC (Figure 2a) was successfully prepared from olive cake with 82.4% of carbon constituents in addition to traces of O, Ca, K, and Si elements ascended from olive cake precursor [2,40]. Oxidation of the AC (Figure 2b) provided active sites for copper linking, where the oxygen content was improved and affected the morphology of the AC surface by increasing its smoothness and enlarging the size of its pores. On the other hand, the effect of functionalization of the Ox-AC with Cu/Cu_2_O/CuO (Figure 2c) was observed on its surface as detected by EDS with a carbon: copper ratio of 66.2:31.9.

#### 3.1.2. Surface Area and Porosity Determination

**Figure 3:**
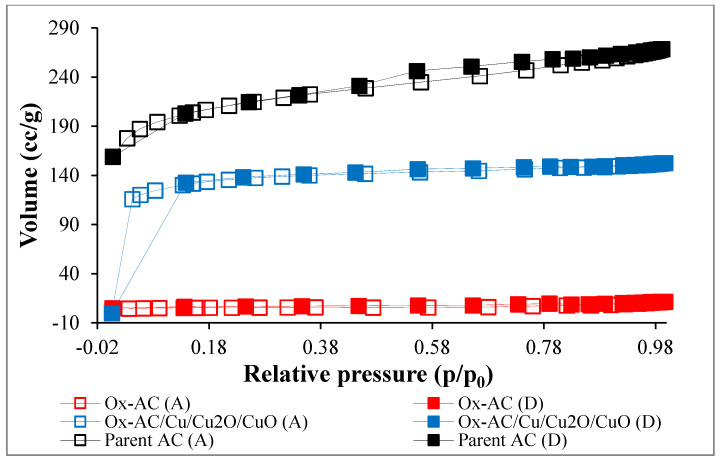
N_2_ adsorption (unfilled points described by letter (A))-desorption (filled points described by letter (D)) isotherms at 77 K for parent AC, Ox-AC, and Ox-AC/Cu/Cu_2_O/CuO.

**Figure 4:**
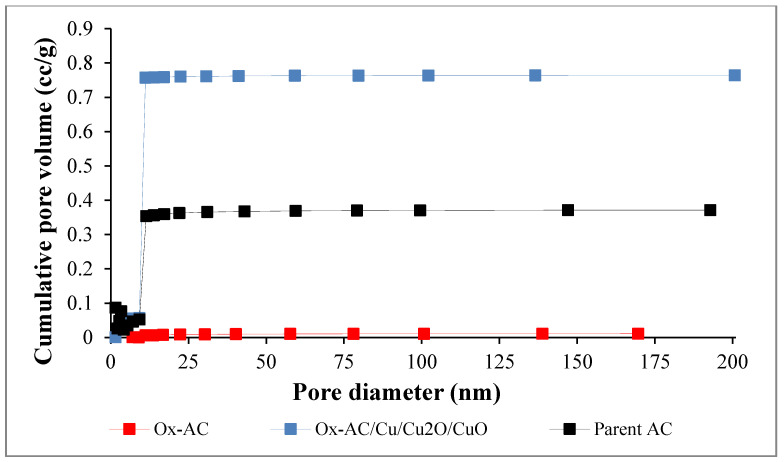
Pores distribution for parent AC, Ox-AC, and Ox-AC/Cu/Cu_2_O/CuO.

The Surface area and porosity properties of the parent AC and functionalized samples are displayed in Figure 3 and Figure 4. The parent AC attained the highest surface area of 697 m^2^/g with mesopores (2 nm < pore size < 50 nm) [41]. However, oxidation of the sample led to a decrease in the surface area to 19 m^2^/g and an increase in the pore size as confirmed also from the SEM image, attaining the mesoporosity size. This may attribute to the blocking of the inner surface area by the oxidized functionalities as the pore volume decreased from 0.414 to 0.018 cc/g after oxidation as presented in Table 2. The low surface area of Ox-AC agrees with the results described by Mines et. al., 2017 [20]. Copper attachment, on the other hand, led to an increase in the surface area to 422 m^2^/g and pore volume to 0.235 cc/g which can be explained by partial re-opening of the blocked pores after oxidation. Although the specific surface area of the Ox-AC/Cu/Cu_2_O/CuO was lower than that of parent AC however, the presence of copper active site will increase the affinity of the adsorbent to phenolic adsorption [42].

#### 3.1.3. Fourier Transform Infrared Spectroscopy (FTIR) Characterization

**Figure 5:**
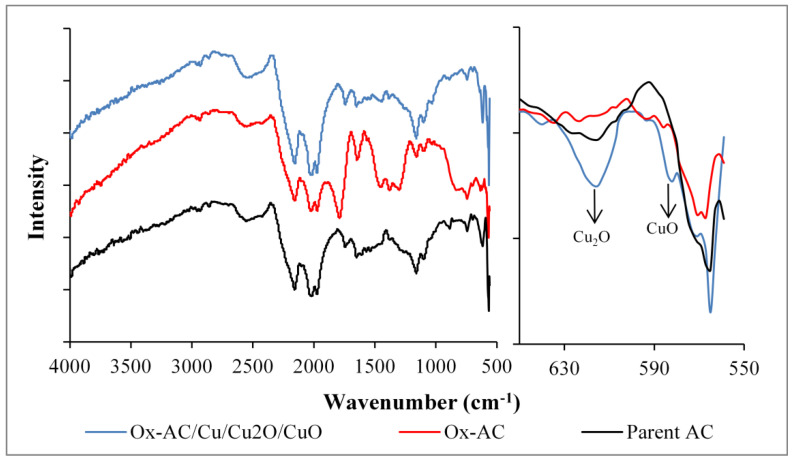
FTIR spectra of parent AC, Ox-AC, and Ox-AC/Cu/Cu_2_O/CuO.

Figure 5 represents the FTIR spectra for parent AC, Ox-AC, and Ox-AC/Cu/Cu_2_O/CuO. The parent activated carbon demonstrate peaks at (2975–2860 cm^−1^), (2500–2400 cm^−1^), (2140–2100 cm^−1^), and (1970–1950 cm^−1^) which suggested the presence of C–H, C=C, C≡C, and C=C=C Allenes functional groups [43]. After the first reaction step with acid oxidation (Ox-AC), three major peaks appeared at (1610–1300 cm^−1^), (1680–1650 cm^−1^), and (1100–1045 cm^−1^) which could be assigned to O=C-O carboxylate stretching, for C=O stretching, and C―O stretching [43]. The spectra of Ox-AC/Cu/Cu_2_O/CuO display two peaks at 580 cm^−1^ and 613 cm^−1^ that could be attributed to Cu-O bonds in both CuO and Cu_2_O, respectively [44].

#### 3.1.4. Point of Zero Charge (pH_pzc_)

**Figure 6:**
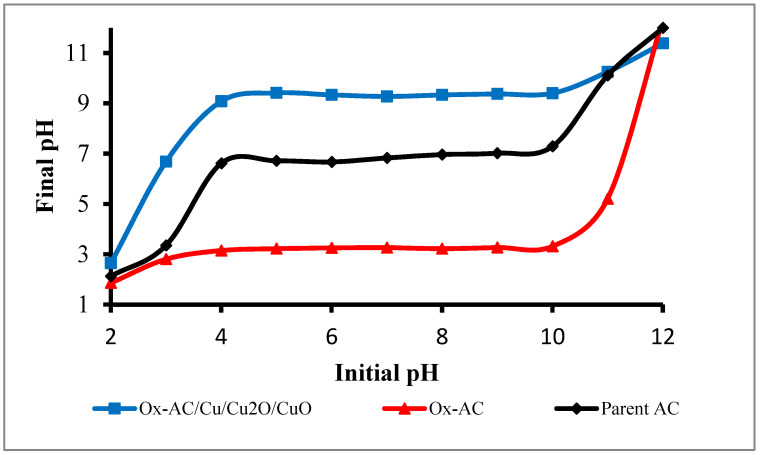
Point of zero charge for parent AC, Ox-AC and Ox-AC/CuO.

The pH drift method was used to determine the point of zero charges (pH_PZC_) for parent AC, Ox-AC, and Ox-AC/Cu/Cu_2_O/CuO. As shown in Figure 6 the pH_PZC_ for parent AC was 7.0 indicating that it comprises carbonaceous material with only aliphatic alkane, alkene, and alkyne functional groups as verified by FTIR results. Oxidation of AC led to the formation of the carboxylic acid group on the carbon surface thus reducing the pH_PZC_ to 3.2. While loading of Cu/Cu_2_O/CuO on its surface leads to an increase in the pH_PZC_ to 9.3, which is compatible with the result attained by Yeddou et al. 2011 [45] since copper oxides have basic characteristics [46].

#### 3.1.5. X-ray Diffraction Pattern (XRD)

**Figure 7:**
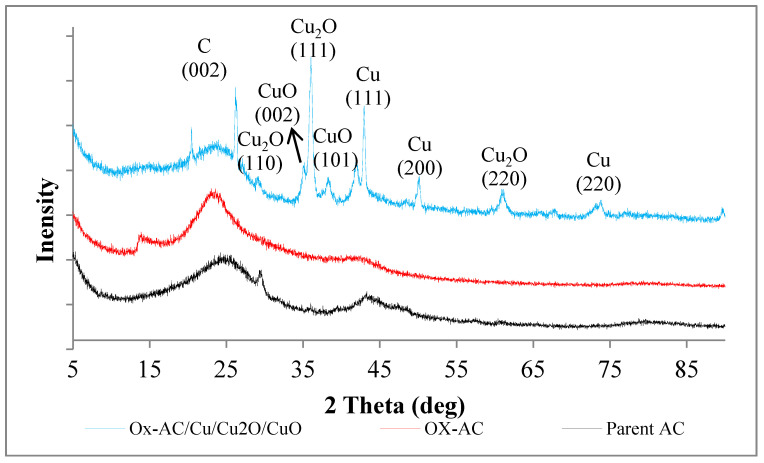
XRD patterns for parent AC, Ox-AC, Ox-AC/Cu/Cu_2_O/CuO.

The presented X-ray diffraction (XRD) for parent AC in Figure 7 exhibits that the AC phase is amorphous. Two peaks at 2θ of 26.4° and 42.8° can be seen for AC. These two peaks are assigned to (002), and (100) planes of AC according to 00-001-0646 reference. The small peak at 28.4° can be assigned to the (111) plane of silicon impurity (00-027-1402 reference) detected by EDS. After oxidation of AC, it was noticed that the two diffraction peaks were slightly increased suggesting that the crystallinity of AC was increased, and shifted to a lower diffraction angle indicating that mesopores were enlarged [47] which was proved also from porosity analysis. Functionalization with copper enhances the crystallinity of AC since the carbon peaks had higher intensity and sharpness. The presence of two peaks at 2θ = 35.1° (002), 38.9° (101) refers to copper oxide (00-001-1117) and the peaks appeared at 2θ = 28.2° (110), 35.9° (111), and 60.8° (220) refers to the diffraction peaks of Cu_2_O [48]. While peaks appear at 42.9°, 50.3°and 73.9° refers to the diffraction peaks of (111), (200), and (220) of metallic Cu (00-002-1225) [49] indicating that AC has a considerable amount of metallic copper besides copper oxides functionality as shown in SEM-EDS.

### 3.2. Treatment of OMW Using Ox-AC/Cu/Cu_2_O/CuO Adsorbent

#### 3.2.1. Influence of Adsorbent Dose

**Figure 8:**
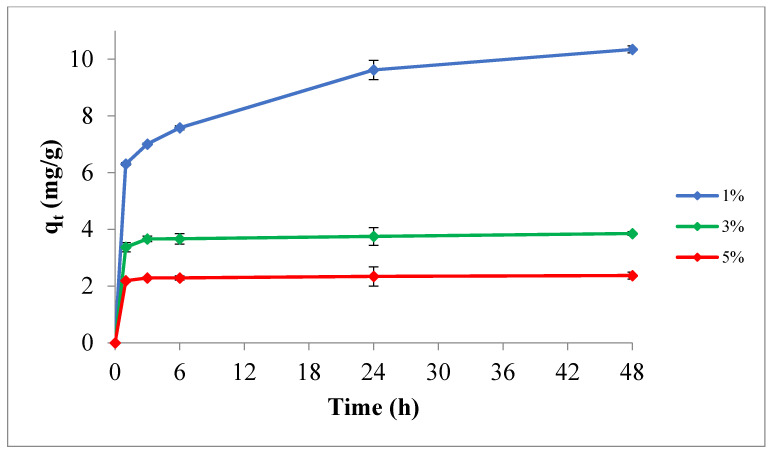
Influence of the adsorbent dose on the adsorption process at temperature of 295 K, pH 5.0, and initial TPC of 124 mg/L.

The effect of the adsorbent dose on TPC uptake, for an initial value of 124 mg/L, temperature of 295 K, and pH of 5.0, is demonstrated in Figure 8. It is obvious that the adsorption process is highly affected by the dose of the adsorbent. Maximum adsorption was 10.2 mg/g for an adsorbent dose of 1%. However, increasing the dose of the adsorbent decreased the equilibrium concentration, which could be attributed to the blocking of certain active sites on the adsorbent surface because of partial aggregation of the adsorbent particles at high concentrations thus decreasing in available surface area for TPC uptake [50,51]. Therefore, increasing the dose beyond 3% or 5% by weight did not enhance the TPC uptake; in contrast, it had a negative impact.

#### 3.2.2. Influence of Contact Time and Initial TPC Concentration

**Figure 9:**
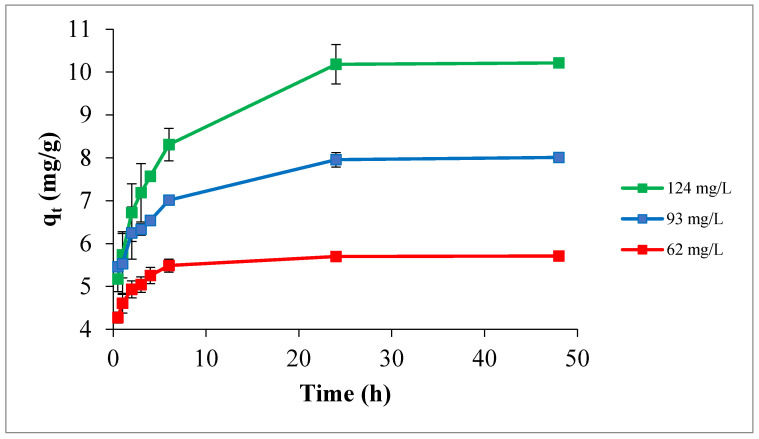
Influence of initial TPC concentration and contact time on adsorption process at temperature of 295 K, pH 5.0, adsorbent dose of 1% by weight.

The influence of initial concentration of TPC and contact time on adsorption was investigated using 1% by wt. Ox-AC/Cu/Cu_2_O/CuO, at a temperature of 295 K, and pH 5.0. Figure 9 shows that the TPC uptake increases with increasing the initial TPC concentration, due to the increase in the mass driving force, which allows more phenols molecules to succeed from the bulk solution to the carbon surface [52]. The curves exhibited a sharp increase of adsorption rate for the three initial TPC concentrations, demonstrating that there are sufficiently accessible sites. Ultimately, at certain values, a plateau was formed indicating that TPC uptake was stopped, and equilibrium was achieved [50,53]. It is obvious from the curves that initial TPC concentrations of 124 and 93 mg/L exhibited similar uptake manners, where the equilibrium was reached after 24 h with equilibrium capacity of 10.2, and 8.0 mg/g, respectively. However, for an initial TPC concentration of 62 mg/L, the equilibrium was reached after 6 h with an equilibrium capacity of 5.7 mg/g.

#### 3.2.3. Influence of the pH

**Figure 10:**
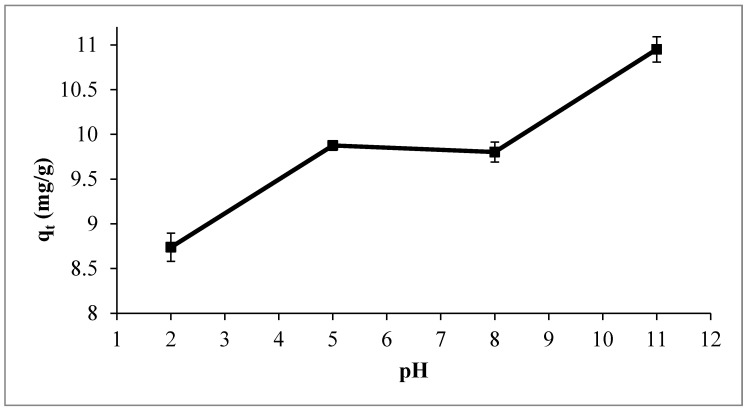
The influence of OMW pH on the TPC uptake using 1% by wt. adsorbent dose, 124 mg/L initial TPC concentration, at temperature of 295 K, and contact time of 24 h.

The pH of the OMW was demonstrated to have a considerable effect on the adsorption capability [50]. Consequently, it is essential to have the best pH value to reach the maximum TPC removal. TPC adsorption as a function of pH was tested in the pH range of 2.0–11.0 with an adsorbent dose of 1% by wt., an initial TPC concentration of 124 mg/L, a temperature of 295 K, and a contact time of 24 h. Figure 10 depicts that the TPC uptake is highly affected by the pH of the OMW; in an extremely acidic medium (pH = 2.0), the adsorption capability for Ox-AC/Cu/Cu_2_O/CuO was comparatively low. This behavior could be attributed to the leaching effect of the copper to copper chloride upon the presence of high concentrations of hydrochloric acid [54,55]. On the other hand, the adsorption efficiency increased in the low pH value, while remaining constant in the pH range of 5–8 may be due to electrostatic attraction between positively charged Ox-AC/Cu/Cu_2_O/CuO (pH_PZC_ = 9.2) and negatively charged deprotonated phenols; the pKa’s for some phenolic compounds were found to be around 4 [56,57,58].

At a highly alkaline solution (pH = 11), the TPC adsorption capacity was elevated. The pH effects cannot be reduced only to a surface charge variation and the adsorption of phenolic compounds must not be comprehended only as of the consequence of electric interactions. Many other interactions can lead to the adsorption of molecules on adsorbent surfaces. This phenomenon could be explained by the electrophilic complexation reaction between phenolate anions and copper metal fixed on the surface of AC where the charge transfer could take place from phenolic groups to the empty d-orbitals of copper since the phenolic compounds are deprotonated at high pH [59]. pH 5 was chosen for subsequent experiments since the pH of raw OMW ranges between 4.5–5; thus, fewer chemicals are required to control the initial pH.

#### 3.2.4. Influence of Ionic Strength

**Figure 11:**
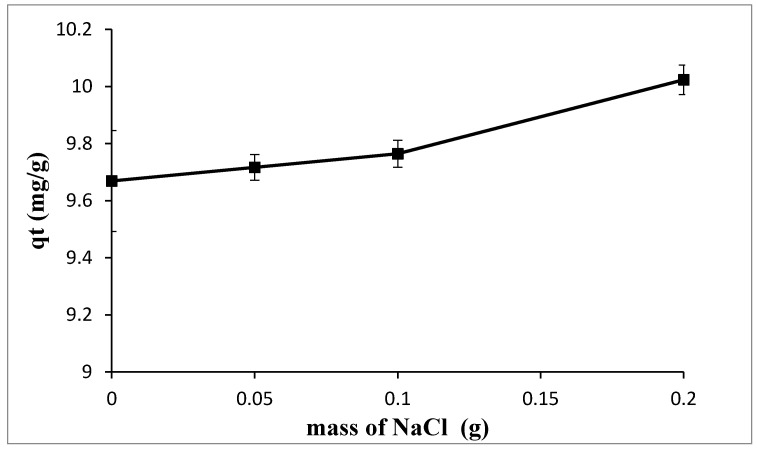
The effect of NaCl addition on adsorption of TPC using 1% by weight adsorbent dose, 124 mg/L initial TPC concentration, at temperature of 295 K, pH of 5.0, and contact time of 24 h.

To evaluate the effect of ionic strength on the adsorption of TPC into Ox-AC/Cu/Cu_2_O/CuO, different sodium chloride masses of 0.00, 0.05, 0.10, and 0.20 g were tested at 295 K, pH 5.0 using 1% by wt. adsorbent dose, and initial TPC concentration of 124 mg/L. As illustrated in Figure 11, the addition of sodium ions has a positive effect on the adsorption of TPC, especially at high concentrations. This phenomenon can be attributed to a salting-out effect; the addition of an electrolyte led to a decrease in the solubility of non-electrolyte, i.e., phenolic compounds thus increasing their diffusion process into the carbon surface [60].

#### 3.2.5. Influence of Temperature and Evaluation of Thermodynamic Parameters

**Figure 12:**
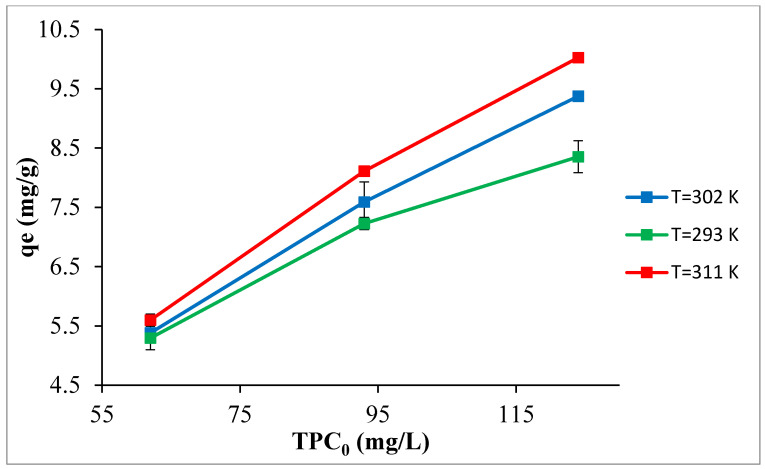
The temperature effect on the TPC uptake using 1% adsorbent dose, pH 5.0, and initial TPC 124 mg/L.

The influence of the temperature on the TPC adsorption is shown in Figure 12. Seemingly, the equilibrium uptake was directly proportional with temperature as the highest TPC uptake was observed at the highest temperature (311K) and the lowest detected at the lowest temperature (293 K). This reveals that the temperature has established a positive effect on the TPC uptake, and consequently, demonstrates the endothermic nature of the process [61]. To estimate the nature of the adsorption process, the thermodynamic parameters such as Gibbs free energy (∆G°), enthalpy change (∆H°), and entropy (∆S°) were evaluated using equations 8, 9, and 10 the plot of the reciprocal of the temperature in K against ln Kc. As presented in Table 3, the enthalpy of the reaction (∆H°) was determined as 30.104 kJ/mol which suggests that the adsorption reaction was endothermic in nature and the removal was due to physical adsorption described by electrostatic interaction between the positively charged adsorbent molecules and the negatively charged phenols, as the magnitude of ∆H° is lower than 40 kJ/mol [51,62,63]. ∆G° was −1.765, −2.839, and −3.723 (kJ/mol) at 293, 302, and 311 K, respectively. The negative sign of the ∆G° indicates that the adsorption process was spontaneous which confirms the feasibility of the process.

### 3.3. Kinetic Study and Reaction Best Adequate Model

**Figure 13:**
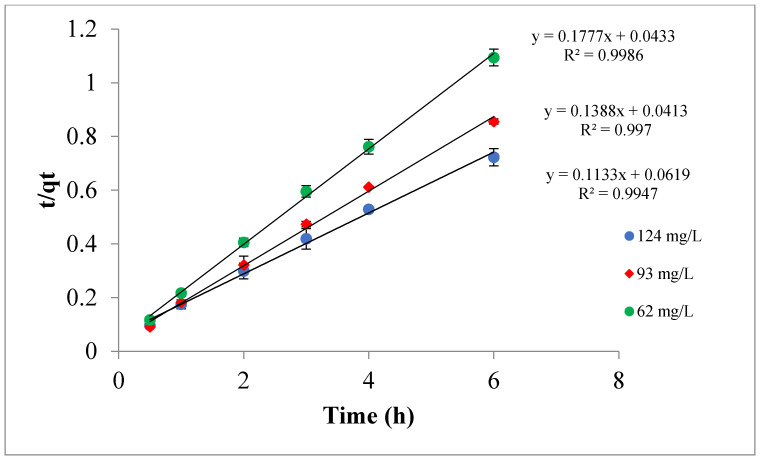
Pseudo-second-order model at pH = 5.0, T = 295 K, and Ox-AC/Cu/Cu_2_O/CuO dose of 1% by wt.

The reaction order and the best fit model were determined from plotting the kinetic data in different reaction models; pseudo-first-order, pseudo second-order, and intraparticle diffusion. The plots of ln(*q_e_* − *q_t_*) and t/*q_t_* versus time were used to test the validity of the pseudo-first-order and pseudo-second-order, respectively, whereas the plot of *q_t_* versus square root of time was used for the diffusion model. The best acceptable model was found to be the pseudo-second-order reaction model; in which straight lines were attained indicating that the adsorption reaction kinetics data can be best characterized by a pseudo-second-order reaction model (Figure 13). On the other hand, the other two models (pseudo-first-order and Intraparticle diffusion) did not demonstrate high linearity. The kinetic and regression parameters along with the sum of the squares of the errors estimated using excel (ERRSQ) of the three models employed are exhibited in Table 4 where the rate constants for the TPC uptake (k_2_) of the pseudo-second-order model were 1.83, 3.36, and 4.10 mg/g·h. for OMW with initial TPC of 124, 93, and 62 mg/L, respectively.

### 3.4. Adsorption Isotherm

**Figure 14:**
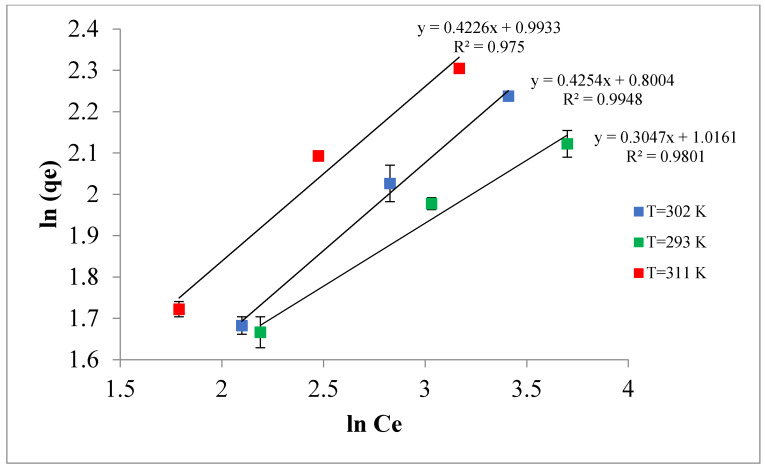
Freundlich linear isotherm representation (adsorbent dose 1% by weight, pH 5.0, contact time 24 h).

**Figure 15:**
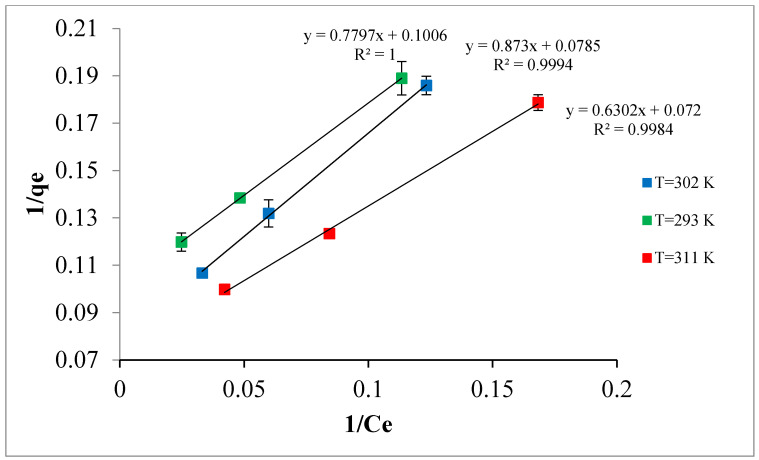
Langmuir linear isotherm representation (adsorbent dose 1% by weight, pH 5.0, contact time 24 h).

The equilibrium data for the adsorption of TPC by Ox-AC/Cu/Cu_2_O/CuO was fitted to the linearized formula of the Langmuir and Freundlich relations as presented in Figure 14 and Figure 15. The values of the calculated parameters along with ERRSQ and regression coefficients at different temperatures are accessible in Table 5. It seems that the Langmuir model perfectly fits the experimental data where the R^2^ values approach one, indicating that the adsorption is limited to one molecular layer where no side interaction between adjacent adsorbed molecules when a single molecule occupies a single surface site [64]. The maximum adsorption capacities, Q_m_, for full monolayer coverage are found at 13.9, 12.7, and 9.9 mg/g for 311, 302, and 293 K adsorption temperatures, respectively.

The major characteristics of raw OMW, OMW after pretreatment with centrifugation and filtration, and OMW after treatment with Ox-AC/Cu/Cu_2_O/CuO at the optimum conditions are ordered in Table 6. The final pH of OMW was increased after treatment with OX-AC/Cu/Cu_2_O/CuO as it has basic characteristics with pH_pzc_ = 9.3. Moreover, the density of OMW was decreased from 1.015 to 1.002. A reduction in the concentration of all polluting parameters was observed after treatment with maximum percent removal of 85%, 42%, 89%, and 88% for TPC, COD, TSS, and TDS, respectively.

## 4. Conclusions

Highly effective activated carbon adsorbent derived from recyclable olive cake and functionalized with copper (Ox-AC/Cu/Cu_2_O/CuO) was prepared for phenolic compounds removal from olive mill wastewater. The uptake of TPC on Ox-AC/Cu/Cu_2_O/CuO increased with increasing the pH of OMW, the initial concentration of TPC, ionic strength, and temperature, while decreased with increasing the adsorbent dose. The adsorption process revealed a high correlation with the Langmuir isotherm model with a maximum TPC adsorption capacity of 13.9 mg/g at 311 K. Kinetic calculations revealed the pseudo-second-order model. In addition, the thermodynamic study demonstrated the spontaneity of the reaction and validated its endothermic nature. The percent uptake of TPC (85%), COD (42%), TSS (89%), and TDS (88%) by the adsorbent product indicates the potential of adsorbent derived from olive cake to be adopted and utilized by olive-mills owners to reduce water pollution and eliminate the environmental problems associated with OMW.

## Figures and Tables

**Table 1:** EDS analysis for parent AC, Ox-AC, Ox-AC/Cu/Cu_2_O/CuO.

Sample	Element %					
	Carbon	Oxygen	Silicon	Potasium	Calcium	Copper
Parent AC	82.4	7.3	0.9	2.8	6.6	-
Ox-AC	75.3	22.1	2.7	-	-	-
Ox-AC/CuO	66.2	1.4	0.5	-	-	31.9

**Table 2:** Surface area (BET), total pore volume, and pore size for parent AC, Ox-AC, and Ox-AC/Cu/Cu_2_O/CuO.

Sample	Surface Area (m^2^/g)	Total Pore Volume (cc/g)	Pore Diameter (nm)
Parent AC	697	0.414	2.38
Ox-AC	19	0.018	3.76
Ox-AC/Cu/Cu_2_O/CuO	422	0.235	2.23

**Table 3:** Thermodynamic parameters evaluated at different temperatures.

TPC_0_ (mg/L)	T(K)	Kc	∆G° (kJ/mol)	∆S° (kJ mol^−1^K^−1^)	∆H° (kJ mol^−1^)
124	293	2.06	−1.765	0.109	30.104
	302	3.10	−2.839		
	311	4.22	−3.723		

**Table 4:** Kinetic parameters, ERRSQ, and regression coefficients for the adsorption of TPC by Ox-AC/Cu/Cu_2_O/CuO at different initial TPC concentrations.

TPC_0_ (mg/L)	First-Order Model			Second-Order Model			Intraparticle Diffusion Model		
	k_1_ (h^−1^)	R^2^	ERRSQ	K_2_ (mg/g·h)	R^2^	ERRSQ	K_p_ (mg/g·h^0.5^)	R^2^	ERRSQ
124	0.18	0.9858	0.9 × 10^−2^	1.83	0.9947	1.4 × 10^−3^	1.80	0.9911	6.1 × 10^−2^
93	0.17	0.9624	2.4 × 10^−2^	3.36	0.997	1.2 × 10^−3^	0.91	0.9642	6.4 × 10^−2^
62	0.35	0.9878	3.2 × 10^−2^	4.10	0.9986	1.0 × 10^−3^	0.67	0.9776	2.1 × 10^−2^

**Table 5:** Freundlich and Langmuir parameters, ERRSQ, and regression coefficients at different temperatures for the adsorption of TPC by Ox-AC/Cu/Cu_2_O/CuO.

Adsorption Temperature (K)	Freundlich Isotherm				Langmuir Isotherm			
	K_F_	*n*	R^2^	ERRSQ	Q_m_	b	R^2^	ERRSQ
311	2.7	2.4	0.9749	4.3 × 10^−3^	13.9	0.11	0.9984	5.1 × 10^−6^
302	2.2	2.4	0.9948	8.2 × 10^−4^	12.7	0.09	0.9994	1.8 × 10^−6^
293	2.8	3.3	0.9801	2.2 × 10^−3^	9.9	0.13	0.9999	4.0 × 10^−^^8^

**Table 6:** The major characteristics of OMW samples.

Parameter	Raw OMW	Reading after Oxidation and Filtration	Reading after Treatment with Ox-AC/Cu/Cu_2_O/CuO
pH	5.5	2.3	6.1
TPC (mg/L)	150	124	22
COD (g/L)	83	65	48
TSS (g/L)	20.67	4.41	2.32
TDS (mg/L)	8630	8350	1004
Density (g/mL)	1.015	1.008	1.002

## Data Availability

Data from this study can be made available upon request.
